# Investigating the effects of cannabinoids for the reduction of inflammation and sickle cell disease pain (CRISP); A protocol for a randomized double-blind placebo-controlled study

**DOI:** 10.1371/journal.pone.0340917

**Published:** 2026-01-28

**Authors:** Jordan Bellis, Lydia Monk, Ritika Jhawar, Galia Pollock, Angela Liu, Charleen Jacobs-McFarlane, Brittany McCrary, Jeffrey Glassberg, Susanna Curtis

**Affiliations:** 1 Department of Emergency Medicine, Icahn School of Medicine at Mount Sinai, New York, New York, United States of America; 2 Department of Hematology and Medical Oncology, Icahn School of Medicine at Mount Sinai, New York, New York, United States of America; 3 Department of Medicine Nursing, Icahn School of Medicine at Mount Sinai, New York, New York, United States of America; PLOS: Public Library of Science, UNITED STATES OF AMERICA

## Abstract

Sickle Cell Disease (SCD) is a hemoglobinopathy affecting millions of people globally. Pain, both acute and chronic, affects over half of those living with SCD, but treatment of chronic pain is an ongoing challenge. While opioid treatments are widely used for chronic pain, it’s efficacy is limited, so alternatives must be explored. This protocol outlines a procedure for investigation of dronabinol, an FDA-approved synthetic tetrahydrocannabinol (THC), for the treatment of pain in patients living with SCD and chronic pain. The study is an 8-week, randomized, double-blind placebo-controlled study which aims to assess both the efficacy and safety of this opioid alternative to pain treatment. The study will also track biomarkers of inflammation as THC has demonstrated anti-inflammatory properties, and inflammation is a driver of SCD pain and disease severity. Results from this study have the potential to further clinical understanding of cannabinoids for pain management in Sickle Cell Disease treatment and spark new questions for research.

## Introduction

Sickle cell disease (SCD) is an inherited blood disorder, characterized by hemoglobin that polymerizes under hypoxic conditions, causing sickling of the red blood cells and leading to chronic hemolysis and vaso-occlusion [1]. Chronic pain, defined as ongoing pain on most days over the past 6 months, affects over half of the 100,000 Americans living with SCD [2,3]. Chronic pain in SCD is multifactorial and can be due to tissue damage, inflammation, neuropathic pain, and peripheral and central nervous system sensitization [1].

Despite the prevalence of chronic pain in patients with SCD, treatment remains challenging. Opioid treatment is the current standard therapy for acute pain but is often also used for chronic pain [3]. However, meta-analysis reveals limited evidence of the efficacy of opioid treatment in relation to its associated risk for treatment of chronic SCD pain [4–7]. There is a critical need for additional agents to treat chronic pain in SCD.

Cannabis is currently being evaluated to treat chronic pain in a myriad of diseases, including in SCD. Cannabis and other cannabinoid products are frequently used among some patients with SCD [8,9]. One survey conducted at an urban, academic medical center treating SCD patients found that 42% of respondents reported marijuana use within the past 2 years, a majority of whom endorsed marijuana use for pain [10]. Another study showed patients living with SCD who obtained medical cannabis at an urban academic medical center had a reduction in the rate of admissions compared to those who requested medical cannabis certification but never obtained it [9]. In a randomized, placebo-controlled study, the use of inhaled cannabis numerically reduced pain and pain interference, as measured by the Brief Pain Inventory, in patients with SCD and chronic pain but the reduction did not meet statistical significance, though improvement in mood did [11]. While some patients with SCD are already using cannabinoid-containing products for pain, the safety, efficacy, and mechanisms of pain relief need further investigation.

Dronabinol is an FDA approved oral agent containing synthetic THC, the primary active ingredient in cannabis, and is a potential agent for pain management in SCD [12,13]. Dronabinol has been shown to be effective for the treatment of chronic pain and neuropathic pain in other diseases [14,15]. It has also been demonstrated to have a possible anti-inflammatory effect [16]. Thus, we hypothesized that dronabinol may be efficacious in treating chronic pain in SCD. To address this hypothesis, we designed a randomized placebo controlled double blinded study which aims to explore the efficacy of dronabinol as an adjunct treatment for chronic pain management in SCD, its potential to improve quality of life and reduce inflammation, and its safety and associated adverse effects [4–6].

## Materials and methods

### Aims

This two-arm randomized, double-blind, placebo-controlled study is designed to test the efficacy of dronabinol, an FDA-approved synthetic tetrahydrocannabinol (THC), as an oral intervention for the treatment of pain in patients living with sickle cell disease and chronic pain.

The trial’s primary outcome is to assess the effect of dronabinol on pain impact, as measured by the Adult Sickle Cell Quality of Life Measurement (ASCQ-Me) Pain Impact score, in adults with sickle cell disease and chronic pain [17,18].

We hypothesize that, compared to the control group receiving placebo, participants randomized to receive the intervention drug will demonstrate an improvement in pain impact at 8 weeks compared to baseline. Additionally, we hypothesize that those randomized to the intervention arm will report an improvement in quality of life and reduction in biomarkers of inflammation.

### Trial design

**Fig 1:**
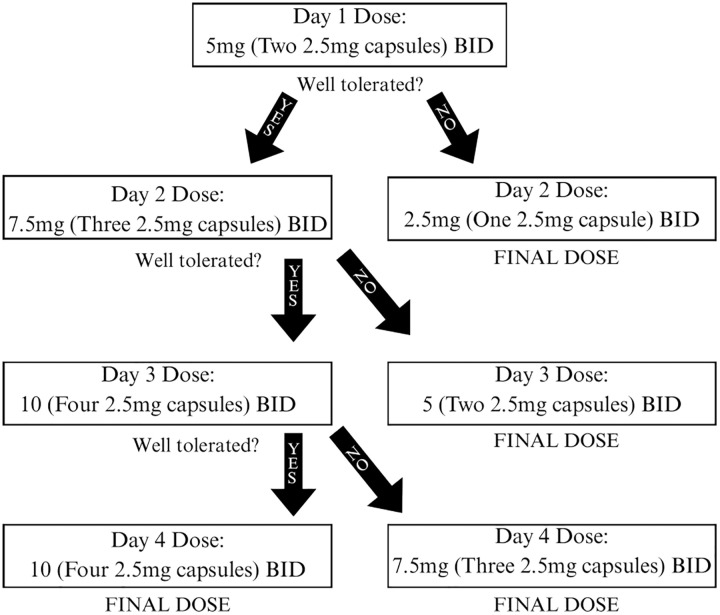
Individualized dronabinol dosing schema.

This study is an 8-week, double-blind, randomized controlled trial. Adults will be randomized to receive oral dronabinol twice daily (treatment arm) or oral placebo (control arm) with a one-to-one allocation at patient individualized doses for 8 weeks (Fig 1). As tolerance to THC varies among individuals, dosing is individualized per patient with the aim to maximize dose while minimizing intolerable side effects [19]. Tolerability is defined by patient preference and the absence of undesirable side effects. Individualized doses will be determined by dose titration over the phone in the first 1–5 days of the study.

### Study setting

Patients will be recruited from the Adult Sickle Cell Program at Mount Sinai Hospital, New York, United States. All study procedures will be undertaken at the Mount Sinai Hospital.

### Eligibility criteria

The clinical research coordinator will consult the study physician to determine if a potential participant is eligible for the trial. If the PI determines that a potential participant is likely eligible and the participant expresses interest in the trial, then the patient will be approached for enrollment (Table 1). Members of the research team including the Principal Investigator (PI) and clinical research coordinators are responsible for assessing whether a potentially eligible patient has the ability to provide written informed consent. Eligible patients will continue on to a screening visit. Screening visits include obtaining informed consent, completing a full eligibility check, and assigning a subject identification number. All study materials will be provided in English.

**Table 1:** Participant eligibility criteria for participation in the CRISP study.

Inclusion Criteria	Exclusion Criteria
Aged 18 years and older	Patient who self-reports daily cannabis use in the past year
All genotypes of sickle cell disease	Patients who are pregnant, breast-feeding, or actively trying to become pregnant
Stable dosages of any SCD-modifying therapy (i.e., hydroxyurea, voxelotor, crizanlizumab, L-glutamine, and/or blood transfusions) as well as any opioids	Patients who are unwilling to use an acceptable form of birth control (i.e., IUD, implants, pills, contraceptives, and true abstinence)
Negative urine toxicology for cannabinoids at least 30 days prior to randomization	Presence or known history of psychotic episode/psychosis or active suicidality (defined by Prodromal Questionnaire Brief and Columbia Suicide Severity Rating Scale respectively)
Patients who can become pregnant must provide a negative pregnancy test at least 30 days prior to randomization	A diagnosis of a current substance-use disorder and/or diagnosis of severe cardiovascular disease in the 6 months prior to consent.
Self-reported pain on most days for 6 months	Unwillingness to avoid use of other cannabis products
ASCQ-Me Pain Impact score ≤ 60	Unable to provide informed consent

### Sreening procedure/randomization

Only individuals who meet the inclusion criteria and do not meet any exclusion criteria will become active trial participants. Participants who fail to meet all inclusion criteria will be notified and withdrawn from the study.

### Randomization

Randomization will be stratified 1:1 by the presence of neuropathic pain, as determined by the Leeds Assessment of Neuropathic Symptoms and Signs (LANSS) and self-reported cannabis usage via a computerized algorithm in REDCap [20].

### Data collection and management

**Fig 2:**
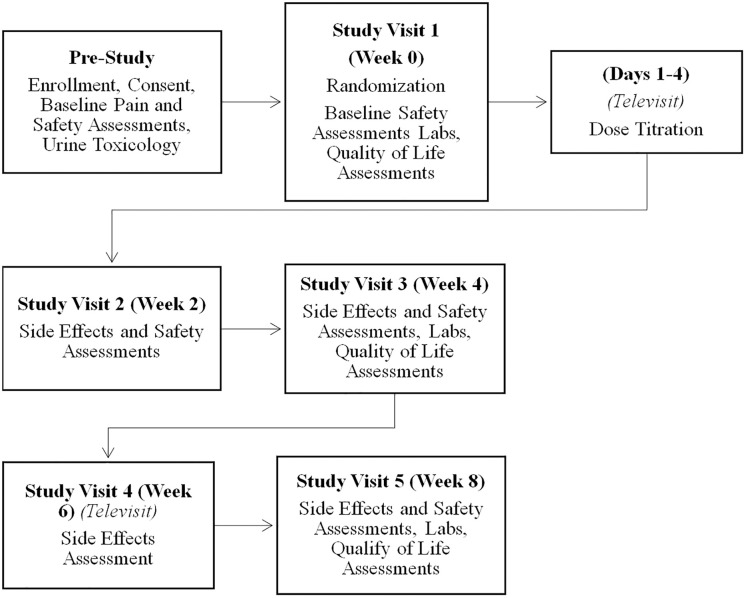
Schedule and flow of study participant visits and assessments.

Demographic data, clinical and patient reported outcomes will be collected at weeks 0, 4, and 8. Safety outcomes and adverse events will be collected at weeks 0, 2, 4, 6, and 8. Prior to randomization, participants will be assigned individualized subject ID numbers so that data can be electronically collected and stored in REDCap without any identifying information (Fig 2).

Patient reported outcomes collected include: Patient-Reported Outcomes Measurement Information System (PROMIS) pain interference (6b), PROMIS neuropathic pain quality (5a), PROMIS nociceptive pain quality (5a), PROMIS anxiety (8a), PROMIS GI nausea and vomiting (4a), PROMIS cognitive function (8a), Adult Sickle Cell Quality of Life Measurement (ASCQ_-Me emotional impact, ASCQ-Me pain impact, ASCQ-Me sleep impact, ASCQ-Me stiffness impact, ASCQ-Me social functioning impact, and ASCQ-Me sickle cell medical history checklist [17,18]. LANSS pain scale will be completed at the enrollment/screening visit and then repeated at visit 3 and 5.

Safety assessments include: The Columbia suicide severity rating scale, Prodromal questionnaire brief will be repeated at visits 2, 3, 4, and 5 (week 2, 4, 6, and 8 respectively), as well as any patient reported adverse effects.

Local laboratory and biomarker assessments will be collected at weeks 0, 4, and 8 and will include complete blood count, reticulocyte count, complete metabolic panel, lactate dehydrogenase, direct bilirubin, tryptase, c-reactive protein, cytokines, VCAM, and P-selectin. Samples collected at weeks 4 and 8 will be sent out for cannabinoid testing to National Medical Services (NMS) Labs.

### Adherence

Patients will be provided with a medication log to record capsules taken each day and any symptoms experienced. For the duration of the study, the clinical research coordinator will routinely record adherence status by counting any remaining study medications that the participant returns as well as totaling the reported number of capsules taken recorded in the medication log. Upon receipt of any study investigational product, the research coordinator will count and return remaining capsules to the research pharmacy. Failure to abstain from other cannabinoid-containing products will be defined as the presence of any minor cannabinoids in the serum present at weeks 4 or 8. To maintain blinding, serum cannabinoid results will not be reviewed by any member of the study team until after the patient has completed the study.

### Sample size and power calculation

A prior study investigated the sensitivity of PROMIS pain impact and determined that changes in chronic pain patients who stated pain was a “little better” had an average 1.8 points with a SD of 6.6 points. Meanwhile, those who reported to be “much better” were associated with an average increase of 6.8 points with a SD of 6.5 points. Based on these positions, we have chosen a change of 5 points to represent a clinically meaningful improvement of chronic pain, with an estimated SD of 6.5 points, for the purposes of this study. A sample size of 28 patients in each group (56 total) will yield 80% power to detect a difference of 5 points for pain impact score from baseline at a two-sided 0.05 significance level between the two groups.

### Analysis

Participant recruitment began in June 2022 and is expected to be completed in February 2027 with final data collection anticipated by May 31^st^, 2027. Results are anticipated in 2027–2028. The primary study outcome is pain impact as defined by the PROMIS pain impact instrument. A difference in differences model will be used to examine the changes from baseline (week 0) through week 8 to compare the effects of dronabinol versus placebo. A linear, mixed effects, repeated measures model will be utilized for measures of inflammation, peripheral blood markers of hemolysis, inflammatory cell populations, and pain scores. Generalized linear models will be applied to healthcare utilization measures.

An initial analysis will be completed using intent-to-treat classification in which all participants who are randomized will be included in the statistical analysis. All randomized subjects, regardless of which treatment they received, are analyzed according to the group they were originally assigned. Secondary analysis will be done omitting subjects who took less than 70% of planned doses as well as those who failed to abstain from other cannabinoid-containing products.

### Oversight and monitoring

The DSMB will meet every 6 months to discuss the study’s progress, approve alterations or amendments to the study protocol, oversee data quality, and review interim analyses. They will also be responsible for monitoring safety and reviewing adverse events (AEs) and serious adverse events (SAEs). AEs will be defined as any temporary unfavorable and unintended symptoms or disease that is associated with the use of a drug. SAEs will include death, hospitalization, life-threatening events, or permanent impairment.

### Ethical considerations

This study protocol has been approved by the Institutional Review Board (IRB). Written, informed consent will be obtained from all participants. Any significant changes to the protocol outlined above (e.g., study design, sample size, or other study aspects) will be written into a formal amendment to be submitted and approved by the IRB before implementation. Participants will be required to be re-consented if any new information results in changes to the consent form.

## Discussion

This study aims to investigate the efficacy of dronabinol, an FDA approved agent containing synthetic THC, for chronic pain management in patients living with SCD. If findings reveal an association between the use of dronabinol and a reduction of chronic pain, this should be examined in a larger study. Further, evaluation of inflammatory biomarkers in study participants throughout the study period will provide data on whether dronabinol has an anti-inflammatory effect in people with SCD. Finally, our study will assess the safety of using an oral THC containing agent in this population.

We chose to examine dronabinol because we believe it may be harm reductive. As an FDA approved oral substance, it is safer than unregulated cannabis. Unregulated cannabis is of variable quality and can be contaminated with harmful substances [21]. Unregulated cannabis is also most-commonly inhaled, but inhaled cannabis is associated with pulmonary toxicity, which can be especially detrimental in SCD [22]. Dronabinol could reduce rates of both unregulated cannabis and opioid use. Participants are asked about prior cannabis use during screening and a urine toxicology test must be negative for the presence of cannabinoids prior to randomization. To examine if subjects use other cannabinoid-containing products during the study, serum samples are sent for mass spectrometry to confirm the presence of minor cannabinoids. We will thus be able to examine if previous cannabis users are able to replace unregulated cannabis use with dronabinol. Dronabinol may be further harm-reductive by offering an alternative to opioids. Cannabinoids have gained traction in the fight against opioid addiction [23]. A 2016 cross-sectional retrospective study found a 64% decrease in opioid use when cannabinoids are used in conjunction with opioid medication in the treatment of chronic pain [24]. However, another study found that among patients with SCD who were certified for medical marijuana, between those who did and did not acquire medical marijuana, no decrease in opioid use was observed [9]. Thus, the potential of dronabinol to reduce the use of two harmful substances, unregulated cannabis and opioids, requires further investigation.

Dronabinol and any THC containing agent poses some safety concerns which need to be examined in people living with SCD. Some of the reported side effects are drowsiness, confusion, and impairment of cognition [23,25]. These are of particular concern in people with SCD already using opioids, which could exacerbate these side effects. However, they may improve if opioid use is reduced by dronabinol or if fatigue and cognition is worsened by chronic pain, which dronabinol may alleviate. To evaluate these risks, we have included validated measures of mood, social functioning, and cognition as outcomes. Another area of concern for high-dose THC is its association with the risk of psychosis. A 2016 meta-analysis found an almost 4-fold increase in risk of developing psychosis for the heaviest users (as defined by the study) and a 2-fold increase for the average cannabis user in comparison to nonusers [26]. We have excluded from the study anyone with a history of psychosis or risk for psychosis or suicidality as defined by the Columbia-Suicide Severity Rating Scale and the Prodromal Questionnaire — Brief Version [27,28]. By doing this we aim for our study to not only evaluate the potential benefits of dronabinol use in people living with sickle cell disease but also its potential harms.

Our study has multiple limitations. First, it is a small, short (8 weeks), single-center study. Additionally, blinding may be compromised as at higher doses THC is psychoactive and patients may recognize these effects [29]. Additionally, we are examining the potential effects of dronabinol on chronic pain in sickle cell disease but are not examining its effects on pain crisis severity or frequency. Larger, longer, multicenter studies should be done to confirm our study findings and answer further questions. Despite these limitations, our study will still lead to important discoveries about the potential benefits of dronabinol as an agent for the treatment of chronic pain in sickle cell disease or for harm reduction.

In conclusion, findings from our study provide an introduction into the understanding of whether dronabinol is an effective treatment for SCD patients with chronic pain. Further research is needed to better understand the long-term effects of dronabinol. Using patient reported questionnaires and blood laboratory results, this study offers clinicians the opportunity to gain critical insight into the potential of dronabinol to improve pain control in adults with Sickle Cell Disease.

## Supporting information

S1 File Protocol. (DOCX)

S2 File SPIRIT 2025 checklist_Manuscript. (DOCX)
