# Supplementary material for: Investigating the effects of cannabinoids for the reduction of inflammation and sickle cell disease pain (CRISP); A protocol for a randomized double-blind placebo-controlled study
Source: PLoS One. 2026 Jan 28;21(1):e0340917. doi: 10.1371/journal.pone.0340917 (PMC12851442; doi:10.1371/journal.pone.0340917)
Supplement: S1 File — (DOCX) [file pone.0340917.s001.docx]

**Dronabinol for the reduction of chronic pain and inflammation in people with sickle cell disease**

**Unique Protocol Identification Number:**

**National Clinical Trial (NCT) Identified Number:**

**Principal Investigator: Susanna Curtis, MD**

**IND/IDE Sponsor: FDA exemption obtained**

**Draft Number: 0.049**

**22 November 2021**

**Summary of Changes from Previous Version:**

| **Affected Section(s)** | **Summary of Revisions Made** | **Rationale** |
| --- | --- | --- |
|  |  |  |
|  |  |  |

**Table of Contents**

[Statement of Compliance 4](#_Toc88485207)

[1 Protocol Summary 5](#_Toc88485208)

[1.1 Synopsis 5](#_Toc88485209)

[1.2 Schema 7](#_Toc88485210)

[1.3 Schedule of Activities (SoA) 8](#_Toc88485211)

[2 Introduction 10](#_Toc88485212)

[2.1 Study Rationale 10](#_Toc88485213)

[2.2 Background 12](#_Toc88485214)

[2.3 Risk/Benefit Assessment 13](#_Toc88485215)

[2.3.1 Known Potential Risks 13](#_Toc88485216)

[2.3.2 Known Potential Benefits 15](#_Toc88485217)

[2.3.3 Assessment of Potential Risks and Benefits 16](#_Toc88485218)

[3 Objectives and Endpoints 17](#_Toc88485219)

[4 Study Design 21](#_Toc88485220)

[4.1 Overall Design 21](#_Toc88485221)

[4.2 Scientific Rationale for Study Design 21](#_Toc88485222)

[4.3 Justification for Dose 22](#_Toc88485223)

[4.4 End of Study Definition 22](#_Toc88485224)

[5 Study Population 23](#_Toc88485225)

[5.1 Inclusion Criteria 23](#_Toc88485226)

[5.2 Exclusion Criteria 23](#_Toc88485227)

[5.3 Lifestyle Considerations 24](#_Toc88485228)

[5.4 Screen Failures 24](#_Toc88485229)

[5.5 Strategies for Recruitment and Retention 24](#_Toc88485230)

[6 Study Intervention 26](#_Toc88485231)

[6.1 Study Intervention(s) Administration 26](#_Toc88485232)

[6.1.1 Study Intervention Description 26](#_Toc88485233)

[6.1.2 Dosing and Administration 26](#_Toc88485234)

[6.2 Preparation/Handling/Storage/Accountability 28](#_Toc88485235)

[6.2.1 Acquisition and accountability 28](#_Toc88485236)

[6.2.2 Formulation, Appearance, Packaging, and Labeling 28](#_Toc88485237)

[6.2.3 Product Storage and Stability 28](#_Toc88485238)

[6.2.4 Preparation 28](#_Toc88485239)

[6.3 Measures to Minimize Bias: Randomization and Blinding 29](#_Toc88485240)

[6.4 Study Intervention Compliance 30](#_Toc88485241)

[6.5 Concomitant Therapy 30](#_Toc88485242)

[6.5.1 Rescue Medicine **Error! Bookmark not defined.**](#_Toc88485243)

[7 Study Intervention Discontinuation and Participant Discontinuation/Withdrawal 31](#_Toc88485244)

[7.1 Discontinuation of Study Intervention 31](#_Toc88485245)

[7.2 Participant Discontinuation/Withdrawal from the Study 31](#_Toc88485246)

[7.3 Lost to Follow-Up 31](#_Toc88485247)

[8 Study Assessments and Procedures 33](#_Toc88485248)

[8.1 Efficacy Assessments 33](#_Toc88485249)

[8.2 Safety and Other Assessments 34](#_Toc88485250)

[8.3 Adverse Events and Serious Adverse Events 35](#_Toc88485251)

[8.3.1 Definition of Adverse Events (AE) 35](#_Toc88485252)

[8.3.2 Definition of Serious Adverse Events (SAE) 36](#_Toc88485253)

[8.3.3 Classification of an Adverse Event 37](#_Toc88485254)

[8.3.4 Time Period and Frequency for Event Assessment and Follow-Up 39](#_Toc88485255)

[8.3.5 Adverse Event Reporting 39](#_Toc88485256)

[8.3.7 Reporting Events to Participants 40](#_Toc88485257)

[8.3.8 Events of Special Interest 40](#_Toc88485258)

[8.3.9 Reporting of Pregnancy 40](#_Toc88485259)

[8.4 Unanticipated Problems 40](#_Toc88485260)

[8.4.1 Definition of Unanticipated Problems (UP) 40](#_Toc88485261)

[8.4.2 Unanticipated Problem Reporting 41](#_Toc88485262)

[8.4.3 Reporting Unanticipated Problems to Participants 41](#_Toc88485263)

[9 Statistical Considerations 42](#_Toc88485264)

[9.1 Statistical Hypotheses 42](#_Toc88485265)

[9.2 Sample Size Determination 42](#_Toc88485266)

[9.3 Populations for Analyses 43](#_Toc88485267)

[9.4 Statistical Analyses 43](#_Toc88485268)

[9.4.1 General Approach 43](#_Toc88485269)

[9.4.2 Analysis of the Primary Efficacy Endpoint(s) 43](#_Toc88485270)

[9.4.3 Analysis of the Secondary Endpoint(s) 43](#_Toc88485271)

[9.4.4 Safety Analyses 44](#_Toc88485272)

[9.4.5 Baseline Descriptive Statistics 44](#_Toc88485273)

[9.4.6 Planned Interim Analyses 44](#_Toc88485274)

[9.4.7 Sub-Group Analyses 44](#_Toc88485275)

[9.4.8 Tabulation of Individual Participant Data 45](#_Toc88485276)

[9.4.9 Exploratory Analyses **Error! Bookmark not defined.**](#_Toc88485277)

[10 Supporting Documentation and Operational Considerations 46](#_Toc88485278)

[10.1 Regulatory, Ethical, and Study Oversight Considerations 46](#_Toc88485279)

[10.1.1 Informed Consent Process 46](#_Toc88485280)

[10.1.2 Study Discontinuation and Closure 46](#_Toc88485281)

[10.1.3 Confidentiality and Privacy 46](#_Toc88485282)

[10.1.4 Future Use of Stored Specimens and Data 46](#_Toc88485283)

[10.1.5 Key Roles and Study Governance 46](#_Toc88485284)

[10.1.6 Safety Oversight 46](#_Toc88485285)

[10.1.7 Clinical Monitoring 46](#_Toc88485286)

[10.1.8 Quality Assurance and Quality Control 46](#_Toc88485287)

[10.1.9 Data Handling and Record Keeping 47](#_Toc88485288)

[10.1.10 Protocol Deviations 47](#_Toc88485289)

[10.1.11 Publication and Data Sharing Policy 47](#_Toc88485290)

[10.1.12 Conflict of Interest Policy 47](#_Toc88485291)

[10.2 Additional Considerations 47](#_Toc88485292)

[10.3 Abbreviations 47](#_Toc88485293)

[10.4 Protocol Amendment History 49](#_Toc88485294)

[11 References 50](#_Toc88485295)

# Statement of Compliance

1. The trial will be carried out in accordance with International Council on Harmonisation Good Clinical Practice (ICH GCP) and the following:
   - United States (US) Code of Federal Regulations (CFR) applicable to clinical studies (45 CFR Part 46, 21 CFR Part 50, 21 CFR Part 56, 21 CFR Part 312, and/or 21 CFR Part 812).

National Institutes of Health (NIH)-funded investigators and clinical trial site staff who are responsible for the conduct, management, or oversight of NIH-funded clinical trials have completed Human Subjects Protection and ICH GCP Training.

The protocol, informed consent form(s), recruitment materials, and all participant materials will be submitted to the IRB for review and approval. Approval of both the protocol and the consent form(s) must be obtained before any participant is consented. Any amendment to the protocol will require review and approval by the IRB before the changes are implemented to the study. All changes to the consent form(s) will be IRB approved; a determination will be made regarding whether a new consent needs to be obtained from participants who provided consent, using a previously approved consent form.

# 1 Protocol Summary

## 1.1 Synopsis

| **Title:** | Dronabinol for the reduction of chronic pain and inflammation in people with sickle cell disease |
| --- | --- |
| **Study Description:** | A randomized, double blind, study of dronabinol as a palliative agent in the treatment of pain, inflammation, and other complications of sickle cell disease (SCD). |
| **Objectives:** | \| Primary Objective: \| To determine whether dronabinol will improve pain and QOL in adults with SCD and chronic pain. \| \| --- \| --- \| \| Secondary Objectives: \| To assess dronabinol’s effect on markers of inflammation in patients with SCD compared to placebo.  To determine the safety and tolerability of dronabinol use in adults with SCD compared to placebo. \| |
| **Endpoints:** | \| Primary Endpoint: \| Change in PROMIS pain impact score \| \| --- \| --- \| \| Secondary Endpoints: \| - ASCQ-Me survey domains for emotional impact, social impact, stiffness, and sleep as well as Patient Reported Outcome Information System (PROMIS) domains for pain impact, neuropathic pain, and nociceptive pain and Leeds assessment of neuropathic signs and symptoms - WBC with differential, CRP, tryptase, substance P and cytokines IL1a, IL1b, IL6, IL4, IL10 and tumor necrosis factor alpha (TNFα). - Self-reported adverse effects, PROMIS domains for anxiety, appetite, nausea, and cognitive function, opioid use in oral morphine equivalents (OME), episodes of emergency room, hospital, or psychiatric facility utilization, Columbia suicide severity rating scale, prodromal questionnaire brief version \| |
| **Study Population:** | - Age >18 years - Clinical diagnosis of SCD (HbSS, HbSC, HbSβ+; Thal, HbSβ0Thal, HbS variants) - Baseline score of 60 or lower on the ASCQ-Me 7-day pain interference domain - If on a SCD modifying therapy (hydroxyurea, regular blood transfusions, L-glutamine, voxelotor, crizanlizumab), on stable dose for at least 3 months - If using opioids for pain at home, on stable dose for at least 3 months - One urine toxicology negative for cannabinoids within 30 days of randomization - No known intolerance to dronabinol, or marijuana - No history of psychotic episode, psychosis, or active suicidality - No contraindication to dronabinol with attention to potential side effects, concurrent medications/substances, and concurrent medical problems, as evaluated by a physician - Willing to abstain from cannabis, medical and illicit, during study weeks 1 through 8 - Not pregnant or nursing - If a woman capable of becoming pregnant, willing to use a medically accepted form of birth control for the duration of study participation.  Accepted forms include oral contraception, medroxyprogesterone, contraceptive implants or patch, surgical sterilization, total abstinence. - Able to consent for research - Daily cannabis use - Diagnosis of active substance use disorder |
| **Phase:** | 2 |
| **Description of Sites/Facilities Enrolling Participants:** |  |
| **Description of Study Intervention:** | Dronabinol or matching placebo |
| **Study Duration:** | 48 months |
| **Participant Duration:** | 8 weeks |

## 1.2 Schema

## 1.3 Schedule of Activities (SoA)

| **Related Aim** | **Related Skill** | **Form** | **Measure** | **Baseline** | | **Week 2** | | **Week 4** | | **Week 6** | | **Week 8** | |
| --- | --- | --- | --- | --- | --- | --- | --- | --- | --- | --- | --- | --- | --- |
| 1 | 1,2,4 | survey | PROMIS Pain Impact | X | |  | | X | |  | | X | |
| 1 | 1,2,4 | survey | ASCQ-Me Pain Impact | X | |  | | X | |  | | X | |
| 1 | 1,2,4 | survey | LANSS Scale | X | |  | |  | |  | | X | |
| 1 | 1,2,4 | survey | PROMIS Pain Quality Neuropathic | X | |  | | X | |  | | X | |
| 1 | 1,2,4 | survey | PROMIS Pain Quality Nociceptive | X | |  | | X | |  | | X | |
| 1 | 1,2,4 | EMR | Daily Opioid Use (OME) | X | |  | | X | |  | | X | |
| 1 | 1,2 | survey | Self Reported Episodes of Pain Crisis | X | |  | | X | |  | | X | |
| **Quality of Life Related Outcomes:** | | | | |  | |  | |  | |  | |  |
| 1 | 1,2 | survey | ASCQ-Me Stiffness | X | |  | | X | |  | | X | |
| 1 | 1,2 | survey | ASCQ-Me Sleep Impact | X | |  | | X | |  | | X | |
| 1 | 1,2 | survey | ASCQ-Me Emotional Impact | X | |  | | X | |  | | X | |
| 1 | 1,2 | survey | ASCQ-Me Social Impact | X | |  | | X | |  | | X | |
| **Inflammatory Markers:** | | | | | | |  | |  | |  | |  |
| 2 | 1,2,3 | blood | White Blood Cell Count | X | |  | | X | |  | | X | |
| 2 | 1,2,3 | blood | Absolute Neutrophil and Monocyte Count | X | |  | | X | |  | | X | |
| 2 | 1,2,3 | blood | Substance P | X | |  | | X | |  | | X | |
| 2 | 1,2,3 | blood | Tryptase |  | |  | |  | |  | |  | |
| 2 | 1,2,3 | blood | Pro Inflammatory: IL1-b, TNFα, IL-6, VCAM-1 | X | |  | | X | |  | | X | |
| 2 | 1,2,3 | blood | Anti Inflammatory: IL4, IL10 | X | |  | | X | |  | | X | |
| **Safety and Tolerability** | | | | |  | |  | |  | |  | |  |
| 3 | 1 | survey | PROMIS Anxiety | X | |  | | X | |  | | X | |
| 3 | 1 | survey | PROMIS GI Nausea and Vomiting | X | |  | | X | |  | | X | |
| 3 | 1 | survey | PROMIS Cognitive Function | X | |  | | X | |  | | X | |
| 3 | 1 | CRA | Self-Reported Adverse Effects | X | | X | | X | | X | | X | |
| 3 | 1 | EMR | Hospital Utilization (ER/Admissions) | X | |  | | X | |  | | X | |
| 3 | 1 | urine | Urine Pregnancy Test | X | |  | | X | |  | | X | |
| 3 | 1 | blood | BMP and liver tests | X | |  | | X | |  | | X | |
| **Other:** | | | | |  | |  | |  | |  | |  |
| 1 | 1 | urine | Urine Toxicology | X | |  | | X | |  | | X | |
| 1 | 1 | CRA | pill count | X | |  | | X | |  | | X | |
| 3 | 1,4 | survey | PQ-B and C-SSRS | X | | X | | X | | X | | X | |

# 2 Introduction

## 2.1 Study Rationale

**Scientific Premise:** People with sickle cell disease (SCD) have chronic pain from multiple sources^2,3,15^ Inflammation is a driver of end organ damage and early mortality in SCD.^16^ Tetrahydrocannabinol (THC) is a partial agonist for cannabinoid receptors (CB1 and CB2), and has been shown to be effective for the treatment of chronic pain and neuropathic pain in conditions such as cancer and multiple sclerosis.^6,17,18^ There is also evidence from murine models of SCD that CB1 agonists can reduce signs of chronic and neuropathic pain as well as inflammatory cytokines that are important in pain development in SCD.^3,4,19^ The proposed research is built on the scientific premise that dronabinol, an FDA-approved synthetic THC medication may treat chronic pain in SCD and reduce inflammation, and therefore is a promising agent for either the treatment of chronic pain or for treatment of the disease directly through reduction of inflammation.

**Agents are needed to target chronic pain in people with sickle cell disease:** The most common symptom of SCD is pain.^20^ In childhood this manifests as pain crises presumed due to vaso-occlusion (VOC).^20^ The average adult with SCD reports pain on more than 50% of days, and chronic pain is one of the strongest predictors of poor quality of life.^21,22^ Chronic pain in SCD, defined by the American Pain Society as pain reported present on most days in the past 6 months, is multifactorial and can be due to tissue damage (nociceptive pain) nerve damage (neuropathic pain) inflammation (inflammatory pain) and increased sensitization to pain.^2,23^ Sensitization to pain in SCD can occur peripherally when peripheral nerves develop reduced thresholds to firing, or centrally when changes in pain processing result in pain feeling more severe and/or being associated with increased levels of fear, depression, or anxiety which makes pain less tolerable.^15,24,25^ Chronic pain in SCD is often treated with opioids, but opioids have risks including overdose, and are often inadequate to manage pain.^21^ Thus, there is a critical need for non-opioid pain treatments in SCD.^26^

**Inflammation is a driver of inflammatory pain and disease severity in sickle cell disease:** SCD is complicated by chronic inflammation due to endothelial damage, ischemia-reperfusion injury, and hemolysis, and this can increase during VOC.^3,7,8^ Inflammation can cause pain directly and is also associated with worse disease severity in people with SCD. Substance P and tryptase are makers of pain sensitization in people with SCD (an experience of pain to lower levels of heat, cold, or pressure stimuli than average).^27,28^ Patients with SCD with greater levels of pain sensitization also have higher levels of pro-inflammatory cytokines IL1b, IL6, and TNFα release in response to painful stimuli.^29^ Substance P and the cytokine IL1b can also directly stimulate pain nerves to fire even in the absence of other stimuli (inflammatory pain).^30^ Both substance P, VCAM-1, and tryptase as well as pro-inflammatory cytokines including IL1b, IL6, and TNFα, have been shown to be elevated at baseline, while anti-inflammatory cytokines such as IL4 and IL10 are reduced at baseline, in people living with SCD, and these differences increase during pain crisis.^28,31^ -34Inflammatory mediators are not only correlated with pain sensitization and are involved in the development of pain crisis, elevated markers of inflammation such as high WBC and specifically neutrophilia are also associated with poor outcomes such as stroke or acute chest and increased risk of early mortality.^35,36^ <Therefore, reducing inflammation in people with SCD may improve inflammatory pain and/or pain sensitization, but may also reduce disease morbidity and mortality.

**THC is effective for treatment of multiple chronic pain types in other diseases, and so has potential in sickle cell disease:** THC acts as a partial agonist on two receptors in the body, cannabinoid receptor 1 (CB1), which is primarily located in the peripheral and central nervous systems and has analgesic activity, and cannabinoid receptor 2 (CB2) which is primarily located in the immune system and has anti-inflammatory activity.^37^ 5,38 9,10 39 The study showed a non-significant trend in pain improvement and a significant improvement in mood.^39^ The authors posit 5 days may not have been long enough to show a change in pain. In my preliminary work, I found that THC-containing medical cannabis use in people with SCD was associated with a reduction in hospital admissions compared to those who were certified for but did not start using medical cannabis.^40^ The 2020 American Society of Hematology guidelines on pain management in SCD recommend that studies be done to evaluate possible benefits and risks of cannabinoids in people with SCD and chronic pain.^1^

**THC may reduce inflammation, an important target for pain and disease modification, in patients with sickle cell disease:** Cannabis use in healthy African Americans is associated with lower levels of TNFα and IL6.^41,42^ Mouse models of SCD treated with CB1 and CB2 agonists had lower white blood cell count (WBC) and neutrophil count both at baseline and during pain crisis, reduced levels of tryptase, substance P and pro-inflammatory cytokines including interleukin (IL) IL1a, IL1b, IL6, and tumor necrosis factor alpha (TNFα). THC is a CB1 and CB2 partial agonists so would be expected to have similar effects, but this has not been studied. If THC reduces inflammation this may reduce pain directly, and additionally may reduce pain crisis frequency or SCD disease severity. No studies have reported the effect of THC on inflammation in patients with SCD.

**The adverse effects of Dronabinol use need to be evaluated in people with sickle cell disease.** Dronabinol has a good safety prolife, the most common adverse events are dizziness, drowsiness, and slowed thinking.^43^ However, there are reports of THC containing agents such as cannabis being rarely associated with serious adverse events such as delirium and hallucinations.^6^ People with SCD have high rates of depression, anxiety, and can develop decreases in neurocognition due to disease in the central nervous system.^44^ It is important to examine the adverse effects of dronabinol specifically in patients with SCD. This is especially true in those patients who are already at risk for slowed thinking due to opioid use.

**Dronabinol is an ideal agent for a controlled study of cannabinoids in sickle cell disease:** In order to determine whether a cannabinoid could be effective for the treatment of pain, a controlled study of a single agent should be done. Dronabinol is a good agent for such a study for several reasons. 1. Dronabinol may treat multiple pain types. Dronabinol is FDA-approved for the treatment of nausea and for appetite stimulation, however it has been shown to be effective for the treatment of multiple pain types.^45^ In a four-week open label study of dronabinol for patients with non-cancer chronic pain (nociceptive, neuropathic, and both), dronabinol was associated with a reduction in pain intensity and pain bothersomeness.^46^ In a study of over 600 patients with multiple sclerosis comparing dronabinol, oral cannabis, and placebo either dronabinol or oral cannabis were both associated with a reduction in pain compared to placebo and dronabinol was associated with fewer adverse effects compared to oral cannabis extract.^47^ Dronabinol may also have a benefit in pain sensitization. Dronabinol reduces pain unpleasantness in studies of provoked pain, which may be explained by reduced processing between the sensory (pain severity) and limbic (emotional aspects of pain) areas of the brain seen in functional MRI studies.^48,49^ Thus, dronabinol may reduce pain impact by direct analgesic effect of nociceptive or neuropathic pain or by reducing pain sensitization and pain unpleasantness. 2. Dronabinol is FDA approved. Dronabinol is carefully regulated, can be dosed accurately in capsules, has known pharmacokinetics. It can be purchased from hospital pharmacies so can be easily obtained for investigator-initiated trials. 3. An oral agent is preferable to a smoke one. Oral cannabinoids are preferable to inhaled for the treatment of chronic pain as they have a longer half-life^50^ and do not cause lung toxicity. Patients with SCD are particularly at risk for lung disease such as asthma, acute chest and pulmonary hypertension, and while no studies have examined the effects of inhaled cannabis on lung toxicity in people with SCD, inhaled tobacco is associated with a 2.6 times higher rate of developing acute chest and a 1.9 times higher rate of acute pain in people with SCD.^51^ Inhaled cannabis is associated with higher rates of cough, wheezing and symptoms suggestive of obstructive lung disease in users without SCD.^51^ -534. If found to be effective and safe, dronabinol will be easy for doctors and patients to use. Unlike illicit or medical cannabis products, dronabinol can be prescribed by physicians, so decisions about how to use it are made between the patient and their physicians. It is also covered by insurance, so patients do not have to pay out of pocket. Finally, it is legal so patients are not at risk for harm from the legal system, this is particularly important for people with SCD because they are a minoritized population that primarily identify as Black which both means they are less likely to be offered effective pain treatment by physicians, and are four times more likely to be arrested for illicit cannabis use despite using at the same rates as other ethnic groups.^54^

## 2.2 Background

A study of people with SCD that showed those who use daily illicit cannabis have fewer hospital admissions than non-users with similar pain severity.^9^  Another study showed that obtaining medical cannabis was associated with a subsequent reduction in hospital admission rates for people with SCD.^40^  Both studies suggested that cannabis may help people with SCD treat their pain. To explore this a pilot study of dronabinol for adults with SCD was conducted. The study was a double-masked placebo-controlled crossover study with two 14-day treatment phases with a 14-day washout in between. Participants were randomized to receive dronabinol in the first phase and placebo in the second, or vice versa.

Recruitment. In the 6 months that it was open, 27 patients were approached during regularly scheduled clinic visits, 24 said they would be interested in such a study, 13 signed consent, and 6 patients were randomized (­­**Figure 2**).  Prior or current cannabis users (medical or illicit) were not more likely than non-users to be interested in the study (87.5% vs 90%, p=0.7), though current cannabis users were non-significantly more likely to consent for the study (61.5% vs 38.5%, p=0.6) and or to be randomized (62.5% vs 20%, p=.2). Current medical cannabis users were also not more likely than non-users to be interested in the study (86% vs 85%, p=0.7), to consent for the study (33% vs 65%, p=0.3), or to be enrolled in the study after consent (50% vs 46%, p=0.7).  Three daily cannabis users were withdrawn before randomization due to being unable to cease cannabis use.  Of the 6 patients who were randomized, 5 were illicit cannabis users and one was accessing medical cannabis, but none were daily users.  Thus, illicit or medical cannabis use did not diminish interest in the study, but daily cannabis use was a barrier to enrollment for some patients.

  Retention. Of the 6 participants who were randomized, 100% presented to all 7 study visits, 100% of planned urine and blood samples were collected, and 100% completed the study.  Urine toxicology studies showed that no participants had THC or other cannabinoid metabolites present in their urine during the washout period or placebo phase, and all participants has cannabinoid metabolites present during the treatment phase. Thus, participants were adherent with study procedures and with avoidance of other cannabinoids.

Masking. Ability to mask dronabinol was also examined as other cannabinoid studies have shown it is difficult to mask psychoactive substances.^55^  Participants were asked if they thought they were receiving dronabinol or placebo at the end of each treatment period and again at the end of the study.  At the end of the first treatment phase, 4/6 (66%) guessed correctly, however at the end of the second treatment phase all 6 guessed correctly.  This suggests that dronabinol can be masked on first exposure to drug or placebo, but the crossover design interferes with ability to mask.

At the end of the study, five of the six participants stated they felt dronabinol was helpful for their pain and requested that dronabinol be prescribed to them after the study. Taken together, though the numbers were small, findings indicate that patients found such a study acceptable and dronabinol to be helpful for their pain. Because daily cannabis use was a barrier to enrollment and washout, in the proposed trial we will exclude daily cannabis users. Dronabinol masking was not feasible during the second phase of the crossover, therefore the proposed trial will not include a crossover design.

## 2.3 Risk/Benefit Assessment

### 2.3.1 Known Potential Risks

The risks of the study include: 1) inconvenience of attending research visits, 2) discomfort associated with research interviews, 3) risks of phlebotomy, 4) confidentiality issues associated with research encounters, and 5) risks related to dronabinol use. These risks will be discussed with potential participants during the informed consent process. Alternatives, including not participating in the study, will be described to participants during the informed consent process.

Inconvenience of attending research visits:

This study requires that subjects participate in face-to-face encounters with a research RA at screening, baseline, every two weeks over the 8-week intervention period. Inconvenience of study participants attending research visits will be addressed by 1) reimbursement for time spent in research encounters ($25 and a two way metro card ($5.50) for each of 4 in personresearch visits, at baseline, 2 weeks 4, and 8); 2) conducting assessments in easily accessible locations, on-site at the Adult Sickle Cell clinics. Additionally, participants will be informed by the research assistant during the informed consent process that they can choose to withdraw from the study at any point.

Discomfort associated with research interviews:

Surveys will assess health status, substance abuse, psychiatric symptoms, medication adherence and other sensitive subjects. It is possible that survey questions could be uncomfortable for participants. Participants will be told that they may choose to stop the surveys or withdraw from the study if they find the questions troubling. Participants also have access to medical and counseling staff at the Adult Sickle Cell clinics.

Risks associated with phlebotomy:

We will collect approximately 30 cc of blood for safety labs and markers of inflammation at baseline, week 4 and week 8. Risks include infection and bleeding. An RN collecting specimens will do so according to standard infection control precautions and will hold pressure on the puncture site until hemostasis is achieved.

Confidentiality issues in research encounters:

Because this project focuses on a controlled substance, confidentiality is a major concern. We will be collecting personal information from participants to facilitate follow up and will be asking questions about sensitive data including illicit drug use, alcohol use, mental health, and health status. Our procedures to maintain confidentiality are addressed below.

Risks related to dronabinol use:

Dronabinol is an FDA approved medication with a known safety profile.  Anticipated side effects are listed here.  Most Common Side Effects include: Dizziness, Euphoria, Paranoid Reaction, Sleepiness, Abnormal thoughts, Abdominal pain, Nausea, Vomiting. Serious Side Effects include: Worsening mental (psychiatric) symptoms. Psychiatric symptoms can worsen in people who have mania, depression, or schizophrenia and who take dronabinol. Dronabinol taken with medicines that cause psychiatric symptoms can worsen psychiatric symptoms. Elderly people who take dronabinol may have a greater risk of having psychiatric symptoms. Problems thinking clearly.  Trouble remembering things, concentrating, have increased sleepiness, or confusion. Elderly people may have a greater risk of having problems thinking clearly. Changes in blood pressure. Dronabinol may increase or decrease blood pressure, especially when patients start taking dronabinol or when the dose is changed. Elderly people, especially those with dementia, and people with heart problems may have an increased risk of changes in blood pressure and an increased risk of falls.

Physical Dependence: is a condition that develops as a result of the body adapting to the response of the drug due to its repeated use. The symptoms of physical dependence show at a certain dose when the continuation of drug use is discontinued in an abrupt/sudden manner or when the drug dose is reduced significantly. This effect has only been evidenced by the termination of drug administration. Physical dependence can develop during chronic therapy with dronabinol capsules and can also develop after chronic abuse of marijuana.

Hazardous Activities

Dronabinol capsules can cause and may impair the mental and/or physical abilities required for the performance of hazardous tasks such as driving a motor vehicle or operating machinery. You should not operate motor vehicles or other dangerous machinery until you are certain that this study medication does not affect you adversely.

Concomitant use of other drugs that cause dizziness, confusion, sedation, or somnolence such as CNS depressants may increase this effect (e.g., barbiturates, benzodiazepines, ethanol, lithium, opioids, buspirone, scopolamine, antihistamines, tricyclic antidepressants, other anticholinergic agents, muscle relaxants). Inform patients not to operate motor vehicles or other dangerous machinery until they are reasonably certain that MARINOL does not affect them adversely.

Concomitant use of other drugs that cause dizziness, confusion, sedation, or somnolence such as Central Nervous System (CNS) depressants may increase this effect (e.g., barbiturates (are central nervous system depressants medication that reduce the activity of nerves causing muscles to relax), benzodiazepines (which is central nervous system depressants medication that causes tiredness and are used to treat anxiety disorders, nervousness, panic disorders, muscle spasms, seizures, sleeplessness etc), ethanol, lithium, opioids, buspirone, scopolamine, antihistamines, tricyclic antidepressants, other anticholinergic agents, muscle relaxants).

Psychiatric risk potential:

Though the case reports suggest the possibility of serious side effects, there is insufficient evidence to establish a causal association between dronabinol and psychiatric symptoms. Given the potential psychiatric risks and the FDA warning, and in order to protect participant safety, we have elected to exclude patients with current bipolar disorder with psychosis, or psychotic disorder; current suicidal ideation; past year suicide attempt; or past year psychiatric hospitalization. Psychiatric monitoring plans and criteria for treatment discontinuation are detailed below (sections E2a and E2b).

Dronabinol is pregnancy category C, and there are no adequate studies for determining infant risk when using varenicline during breastfeeding.

### 2.3.2 Known Potential Benefits

Dronabinol has been shown to have benefits for chronic pain in other diseases.  A reduction in chronic pain for adults with sickle cell disease could lead to reduced opioid use, improved quality of life, or both.  It could also lead to reduced hospital utilization.  Other THC containing agents have been associated with reduced markers of inflammation in other diseases and in murine models of sickle cell disease.  Reduced levels of inflammation could lead to reduce pain as well as reduced rates of vaso-occlusive pain crisis and possible reduced end organ damage.

### 2.3.3 Assessment of Potential Risks and Benefits

Given the high prevalence of cannabis use in sickle cell disease, it is important to evaluate both the potential benefits to pain and quality of life and the potential risks. This study could provide new insights into whether THC containing agents should be used by people with sickle cell disease. Should dronabinol prove efficacious for the improvement of pain or quality of life in people with sickle cell disease, or for the reduction of markers of inflammation, we will write an R01 proposal for a phase III multicenter study.  If dronabinol is associated with poor safety or tolerability, this will be important to patients and clinicians in considering if people with sickle cell disease should use other cannabis containing products. As chronic pain in sickle cell disease is one of the largest predictors of poor quality of life, this study is timely and addresses an important public health need.

# 3 Objectives and Endpoints

| OBJECTIVES | ENDPOINTS | JUSTIFICATION FOR ENDPOINTS |
| --- | --- | --- |
| Primary | | |
| Patient Reported Outcome Information System (PROMIS, a non-disease specific patient reported outcome survey system) pain impact score | Change from baseline to end of week 8 | The primary outcome is the PROMIS pain impact score, which assesses not only the severity of the pain but the impact of the pain on the participant’s daily living, a more relevant patient-centered outcome. PROMIS pain impact score has been validated as a onetime measure in people with SCD, and further has been validated to detect changes over in other diseases of chronic pain.^63,64^ |
| Secondary | | |
| Other Patient Reported Outcomes (ASCQ-Me survey domains for emotional impact, social impact, stiffness, and sleep) PROMIS domains for neuropathic pain, nociceptive pain, cognitive function, gastrointestinal distress and anxiety.^66^ LANSS, PD-B, C-SSRS | Change from baseline to end of week 8 | Patients will complete ASCQ-Me survey domains for emotional impact, social impact, stiffness, and sleep as well as Patient Reported Outcome Information System (PROMIS, a non-disease specific patient reported outcome survey system) domains for pain impact, neuropathic pain, nociceptive pain, cognitive function, gastrointestinal distress and anxiety.^66^ Patients will complete the Leed’s Assessment of Neuropathic Symptoms and Signs (LANSS_ at the beginning and end of study. LANSS is a validated scale of pain quality questions with a scale of 0-24 in which a score of 12 or more defines the presence of neuropathic pain which has been previously used to define neuropathic pain in patients with SCD.^67,68^ The Prodromal Questionaire - Brief is a short psychiatric survey validated to assess for early symptoms of psychosis and the Columbia Suicide Severity Rating Scale (C-SSRS) is a validated measure of suicidality.^59,60^ Both will be administered at each office visit. |
| Opioid Consumption in average daily oral morphine equivalents | Change from baseline to end of week 8 | Opioid use will be calculated using average daily morphine-milligram equivalents based on medications dispensed as documented in the New York State electronic prescribing monitoring program. |
| Laboratory Measures of Inflammation: WBC with differential, CRP, tryptase, substance P, VCAM-1 and cytokines IL1a, IL1b, IL6, IL4, IL10 and tumor necrosis factor alpha (TNFα) | Change from baseline to end of week 8 | SCD is complicated by chronic inflammation due to endothelial damage, ischemia-reperfusion injury, and hemolysis, and this can increase during VOC.^3,7,8^ Inflammation can cause pain directly and is also associated with worse disease severity in people with SCD. Substance P and tryptase are makers of pain sensitization in people with SCD (an experience of pain to lower levels of heat, cold, or pressure stimuli than average).^27,28^ Patients with SCD with greater levels of pain sensitization also have higher levels of pro-inflammatory cytokines IL1b, IL6, and TNFα release in response to painful stimuli.^29^ Substance P and the cytokine IL1b can also directly stimulate pain nerves to fire even in the absence of other stimuli (inflammatory pain).^30^ Both substance P, and tryptase as well as pro-inflammatory cytokines including IL1b, IL6, VCAM-1, and TNFα, have been shown to be elevated at baseline, while anti-inflammatory cytokines such as IL4 and IL10 are reduced at baseline, in people living with SCD, and these differences increase during pain crisis.^28,31^ Inflammatory mediators are not only correlated with pain sensitization and are involved in the development of pain crisis, elevated markers of inflammation such as high WBC and specifically neutrophilia are also associated with poor outcomes such as stroke or acute chest and increased risk of early mortality.^35,36^ Therefore, reducing inflammation in people with SCD may improve inflammatory pain and/or pain sensitization, but may also reduce disease morbidity and mortality.  Mouse models of SCD treated with CB1 and CB2 agonists had lower white blood cell count (WBC) and neutrophil count both at baseline and during pain crisis, reduced levels of tryptase, substance P and pro-inflammatory cytokines including interleukin (IL) IL1a, IL1b, IL6, and tumor necrosis factor alpha (TNFα). THC is a CB1 and CB2 partial agonists so would be expected to have similar effects, but this has not been studied. If THC reduces inflammation this may reduce pain directly, and additionally may reduce pain crisis frequency or SCD disease severity. |
| Tertiary/Exploratory | | |
| Analysis as above repeated examining patients with neuropathic pain only, and other pain types only, and patients who were cannabinoid naïve vs previous cannabinoid users, though these may be underpowered. | Change from baseline to end of week 8 | In a four-week open label study of dronabinol for patients with non-cancer chronic pain (nociceptive, neuropathic, and both), dronabinol was associated with a reduction in pain intensity and pain bothersomeness.^46^  In a study of over 600 patients with multiple sclerosis comparing dronabinol, oral cannabis, and placebo either dronabinol or oral cannabis were both associated with a reduction in pain compared to placebo and dronabinol was associated with fewer adverse effects compared to oral cannabis extract.^47^  Dronabinol may also have a benefit in pain sensitization.  Dronabinol reduces pain unpleasantness in studies of provoked pain, which may be explained by reduced processing between the sensory (pain severity) and limbic (emotional aspects of pain) areas of the brain seen in functional MRI studies.^48,49^ Thus, dronabinol may reduce pain impact by direct analgesic effect of nociceptive or neuropathic pain or by reducing pain sensitization and pain unpleasantness. |

# 4 Study Design

## 4.1 Overall Design

Randomized, placebo-controlled, double masked, study of twice daily dronabinol given at a patient-optimized dose for 8 weeks. Participants will be titrated to optimized individual dose during the first week and continue at that dose for the remaining 8 weeks.  Randomization will be stratified by presence/absence of neuropathic pain and by history of cannabis use(yes/no) in blocks of four). Visits will occur every 2 weeks. Primary outcome is a difference in differences analysis of pain impact from baseline to 8 weeks compared between patients on dronabinol and on placebo. 

## 4.2 Scientific Rationale for Study Design

People with sickle cell disease (SCD) have chronic pain from multiple sources^2,3,15^ Inflammation is a driver of pain, end organ damage and early mortality in SCD.^16^ Tetrahydrocannabinol (THC) is a partial agonist for cannabinoid receptors (CB1 and CB2), and has been shown to be effective for the treatment of chronic pain and neuropathic pain in conditions such as cancer and multiple sclerosis.^6,17,18^ There is also evidence from murine models of SCD that CB1 agonists can reduce signs of chronic and neuropathic pain as well as inflammatory cytokines that are important in pain development in SCD.^3,4,19^ The proposed research is built on the scientific premise that dronabinol, an FDA-approved synthetic THC medication may treat chronic pain in SCD and reduce inflammation, and therefore is a promising agent for either the treatment of chronic pain or for treatment of the disease directly through reduction of inflammation.

Randomization will be done 1:1 to dronabinol or placebo in blocks of four, stratified by pain type (neuropathic or not) and past-year cannabinoid use (yes or no). Randomization will be done by the pharmacy. Neuropathic pain will be defined by LANSS score >12. Past-year cannabis use will be defined by self-report of any cannabinoid use (medical, illicit, or cannabidiol containing (CBD) products) in the past year. Based on our prior experience as well as the experience of others, we expect 50% to have neuropathic pain and 50% to have past-year cannabis use after excluding frequent cannabis users.^9,40,61^

The treatment duration will be 8 weeks. Studies of cannabis for the improvement of neuropathic pain in other diseases have ranged in duration from 2 to 26 weeks. Another important component of chronic pain in SCD is chronic inflammation. Changes in inflammatory markers can also take weeks to occur, in studies of acute infections markers of inflammation such as CRP can take up to 6 weeks to normalize.62 Therefore, a study aimed at improving chronic pain in SCD or reducing inflammation should be done over weeks to months. We have chosen 8 weeks which we expect will be long enough to detect efficacy in pain and inflammatory outcomes and identify adverse effects.

In a previous pilot study with a cross over study design ability to mask dronabinol was examined as other cannabinoid studies have shown it is difficult to mask psychoactive substances.^55^ Participants were asked if they thought they were receiving dronabinol or placebo at the end of each treatment period and again at the end of the study.  At the end of the first treatment phase, 4/6 (66%) guessed correctly, however at the end of the second treatment phase all 6 guessed correctly. This suggests that dronabinol can be masked on first exposure to drug or placebo, but the crossover design interferes with ability to mask. Dronabinol masking was not feasible during the second phase of the crossover, therefore this trial includes a placebo arm for does not include a crossover design.  

## 4.3 Justification for Dose

Dronabinol is an FDA approved drug with a good safety profile, known pharmacokinetics, and an oral formulation which is easily mimicked in a placebo formulation. Dosing. Dronabinol will be dosed twice daily. Dronabinol is eliminated in a biphasic manner, with an initial half-life of 4 hours and a terminal half-life of 25 to 36 hours.^6,50^ Decision to use twice daily dosing at an individualized dose is based both on the biphasic half-life and the dosing recommendations for appetite stimulation for which it is FDA approved. Dose titration. The dose of dronabinol will be individualized for each participant during the first week of treatment, from a minimum of 5 mg twice daily to a maximum of 10 mg twice daily. The initial dose for all participants will be 5 mg twice daily.,If it is well tolerated the dose will be increased to 10 mg twice daily. If they do not experience adverse effects, the final dose will be 10 mg twice daily. Placebo capsules will be labelled as 5.0 mg containing matching methylcellulose containing capsules.  As the study will be double-masked, dose optimization will be completed during the placebo arm as above.

Initial study doses ranged from 2.5mg twice daily to 10mg twice daily given in 2.5mg containing capsules. However, 2.5 mg capsules are no longer manufactured and only 5mg capsules are available, so the study has been modified to use 5mg capsules in doses range from 5 to 10 mg twice daily.

## 4.4 End of Study Definition

A participant is considered to have completed the study if he or she has completed all phases of the study including the last visit or the last scheduled procedure shown in the Schedule of Activities (SoA), Section 1.3

# 5 Study Population

## 5.1 Inclusion Criteria

| - Sickle cell disease (all genotypes) - Age >18 - Significant pain impact as defined by ASCQ-Me Pain impact score <60 - Chronic pain as defined by self reported daily or near daily pain in the past 6 months - If on a SCD modifying therapy (hydroxyurea, regular blood transfusions, L-glutamine, voxelotor, crizanlizumab), on stable dose for at least 3 months - If using opioids for pain at home, on stable dose for at least 3 months - Urine toxicology negative for cannabinoids at least 30 days before randomization |
| --- |

## 5.2 Exclusion Criteria

| - Pregnant or breastfeeding - Unwilling to use acceptable birth control - Unable to consent for research - Unwilling to avoid other cannabis products - Daily cannabis use - History of psychotic episode, psychosis, or active suicidality - Diagnosis of substance use disorder - Severe cardiovascular disease (MI, PTCA, unstable angina, CABG, and/or serious arrhythmia in the previous 6 months |
| --- |

## 5.3 Lifestyle Considerations

Subjects must avoid using all cannabinoid containing substances throughout the study period. Subjects who endorse cannabinoid use other than dronabinol should consider early withdrawal from the study.

## 5.4 Screen Failures

Screen failures who fail due to positive urine test for cannabinoids may rescreen up to 3 times at least 2 weeks from date of last screen failure.

Screen failures who fail due to lack of chronic pain or insufficient pain impact score may rescreen in 3 months from date of screen failure.

## 5.5 Strategies for Recruitment and Retention

**Recruitment strategy.** Recruitment will be active and passive. Active recruitment will focus on contacting potentially eligible adults with SCD who are treated at Mt. Sinai’s outpatient and inpatient practices and inviting them to participate. We will employ the following recruitment strategies:

1. Referrals from providers. Patients who are seen for in-person or telemedicine visits with providers in the Sickle Cell Program and are potentially eligible will be referred to study coordinators.
2. Advertising in clinical area. Flyers and brochures about the study will be posted in facilities where adults with SCD are treated
3. Community outreach. We will engage community-based organizations and community leaders to optimize community outreach including events such as health fairs and Sickle Cell Awareness Day events and via advertising the study over email and social media. This will be facilitated through existing partnerships with the Sickle Cell Thalassemia Patients Network and the New York chapter of the Sickle Cell Association of America.

**Feasibility of recruitment.** Recruitment will be feasible. We will primarily recruit patients treated for SCD at the Mount Sinai Adult Sickle Cell Center. Approximately 40 unique patients are seen in clinic each month.  Of approximately 450 adults with SCD treated at the Center we estimate half (225) will have chronic pain, of those 25% will be frequent cannabis users and this ineligible for our study (approximately 56) so we estimate we will have approximately 169 eligible patients. However, we have also created a plan for regularly monitoring recruitment and implementing a contingency plan of expanding to additional NYC Sickle Cell programs if needed.

**Recruitment timeline.** We will recruit for 36 months (see Project Timeline), enrolling 2-3 subjects per month. Recruitment will be feasible; based on the numbers above and prior studies, we expect to identify 30-45 potentially eligible individuals per month, screen 33% of those (10-15), and enroll 20% of these screened (2-3 per month). To monitor progress towards prespecified recruitment benchmarks will be examined at each quarter (3m period).

**Recruitment monitoring.** We will monitor recruitment and enrollment every week using flowcharts of potential subjects who are identified, screened, enrolled, randomized, and certified for medical cannabis. As we do for our other studies, we will review these data at weekly research meetings of the study team, discuss barriers or issues, and institute corrective measures if needed in an ongoing way, such as increasing outreach, letters, and telephone calls. In addition to routine weekly monitoring, we will assess our progress against the specific benchmarks on the schedule described in the Table above, first after 3 months then every 6 months. Recruitment reports will be included in reports to the Data and Safety Monitoring Committee (DSMC) every 6 months.

**Contingency plan.** We expect to meet benchmarks at Mt. Sinai. However, if enrollment falls below 75% of targets in two subsequent quarters, we will activate our contingency plan of recruiting from other New York City Sickle Cell centers. This plan is feasible due to membership in the American Society of Hematology (ASH) Research Consortium Clinical Trials Network of New York City. It is premature to describe details of possible expansion to additional sites.

# 6 Study Intervention

## 6.1 Study Intervention(s) Administration

### 6.1.1 Study Intervention Description

Dronabinol is a synthetic form of 9-D-tetrahydrocannabinol (THC), the most common cannabinoid found in most strains of marijuana. 37 Dronabinol binds both of the known receptors of the endocannabinoid system, CB1, which is located primarily in the nervous system, and CB2, which is located primarily on immune cells.19,38 Dronabinol is a commercially available, Schedule III medication that is currently FDA approved for the treatment of chemotherapy induced nausea and vomiting and for appetite stimulation in patients with AIDS.37,39-41 As such, legal use of dronabinol in research is feasible; indeed, one of us has published a study of dronabinol effects in an human experimental pain model. 42 Dronabinol has been or currently is being studied for control of pain, including neuropathic pain, in diverse disorders, and control of inflammation, especially in inflammatory neurological and gastro-intestinal disorders.17,20,43-48 Although patient blinding is imperfect due to subject recognition of euphoric effects, in many of these studies a placebo- controlled design has resulted in interpretable results.

The schedule is dronabinol/placebo twice daily (approximately upon awakening and 8-12 hours later). Doses should be taken with consistent timing but without regard to meals. All doses will be administered using dronabinol 5 mg/placebo capsules. The starting dose in all subjects is dronabinol/placebo is 5 mg twice daily up to a total of 10 mg a day. Study agent will be dispensed in quantities adequate for two weeks of dosing. All discussions with subjects about dosing will be conducted in terms of numbers of capsules, not mg amounts.

### 6.1.2 Dosing and Administration

Dose titration will be undertaken to identify a subject-specific maximum well tolerated dose (MWTD). MWTD for dose titration will be defined as the maximum dose at which the subject feels there are no intolerable adverse effects.  On Days 2-4, a study team clinician (doctor or nurse) will initiate telephone or face to face contact with the subject to review the dosing goal – identification of a MWTD -- and then determine an agreed-on dose going forward based upon the subject’s experience to date.

**Dosing Schema:**

Day 1: Dronabinol/placebo 2 capsules twice daily.

Day 2: All subjects will have been on a dose of one capsules twice daily.

Options:

- Dose escalate to two capsules twice daily, or
- Withdraw from study if dose is intolerable

Day 3: Subjects will have been on a dose of two capsules twice daily or one capsule twice daily.

Options for subjects on two capsules twice daily:

- - Stay at two capsules twice daily
  - Return to one capsule twice daily.

Options for subjects on one capsule twice daily:

- - Declare one capsule twice daily the MWTD, or
  - Discontinue subject as dronabinol

Upon declaration of a MWTD, subjects will be instructed not to exceed the MWTD dose. Subjects will be encouraged to continue at the MWTD for the duration of the dosing period, but not at the expense of unpleasant side effects, in which case subjects may choose to take less. Subsequent upward adjustments of the declared MWTD, not to exceed four capsules twice daily, are permissible when agreed upon by a subject and study team clinician.

## 6.2 Preparation/Handling/Storage/Accountability

### 6.2.1 Acquisition and accountability

### 6.2.2 Formulation, Appearance, Packaging, and Labeling

### 6.2.3 Product Storage and Stability

### 6.2.4 Preparation

## 6.3 Measures to Minimize Bias: Randomization and Blinding

The study will be double blind, neither investigators or subjects will know whether subjects are receiving dronabinol or placebo during either dosing period. To protect this blinding study team personnel will not be aware of the results of the urinary toxicology done after randomization. Study team personnel and subjects will be aware that subjects will take dronabinol 5 mg capsules or placebo, but they will not know which dosing period involves dronabinol or placebo.

Study group assignment procedures, and rationale for intervention’s dose, frequency and administration.

Subjects meeting all eligibility criteria will be randomized to dronabinol or placebo for 8 weeks.  We will randomize in a 1:1 ratio, via central, computer-generated randomization done by the pharmacy, stratified by history of prior cannabis use (yes or no) and presence of neuropathic pain (yes or no). To minimize bias in treatment group allocation, the data manager will store the allocation sequence using a password-protected file and has no contact with study participants. We will use urn randomization to minimize risks of imbalance in treatment allocation.

Neuropathic pain will be defined by Leeds Assessment of Neuropathic Signs and Symptoms (LANSS) (score >12). Past-year cannabis use will be defined by self-report of any cannabinoid use (medical, illicit, or cannabidiol containing (CBD) products) in the past year. Based on our prior experience as well as the experience of others, we expect 50% to have neuropathic pain and 50% to have past-year cannabis use after excluding frequent cannabis users.

Capsules of generic dronabinol will be over-encapsulated with methylcellulose filler in between the capsules and placebo will be formulated with matching cellulose capsules filled with methylcellulose. Matching labels for dronabinol and placebo bottles will be provided.

Encapsulated Dronabinol Capsules

For blinding, one intact active dronabinol capsule will be placed in a capsule shell, back filled with sufficient quantity of microcrystalline cellulose, and closed. The capsules are visually inspected and packed into Polypropylene bottles .

Placebo for Encapsulated Dronabinol Capsules

Matching placebo will be compounded, using a matching empty capsule filled with sufficient quantity of microcrystalline cellulose. The capsules are visually inspected and packed into Polypropylene bottles.

Unblinding may be done in the case of some serious adverse events (SAEs), defined as those which result in death, are life-threatening, require inpatient hospitalization or prolongation of existing hospitalization, result in significant disability or incapacity, or result in congenital anomaly or birth defect, Dr. Curtis will be notified and if the event is determined to be possibly related to dronabinol it will be discontinued.  Dronabinol will not be discontinued for SAEs determined to be unrelated to dronabinol, such as pain crisis. In the event that a participant withdraws from the study or discontinues dronabinol due to a SAE, the participant will be unblinded.

**Study Intervention Compliance**

**Measures of Adherence:** Blood toxicology and pill count results will be used to determine study drug adherence (both) and adherence to avoidance of other cannabinoid containing substances (blood only) and will be used during secondary non-intention to treat analysis of the data as described below.

**Pill Counts:**Patients should bring medication bottles to visits at weeks 2, 4 and 8.  Pills counts will be done at each visit and compared to pharmacy dispensing records.

**Blood measure of non-dronabinol cannabinoid use:**  Blood cannabinoids test will be done for multiple cannabinoids including THC, cannabidiol (CBD), and others.  This can distinguish 3 states: (1) recent dronabinol alone in which only THC will be present, (2) recent cannabis use, without or with dronabinol, in which multiple cannabinoids will be present and (3) no recent cannabis use in which no cannabinoids will be present.  This was shown to be able to distinguish dronabinol use from other illicit and medical cannabis use in our prior feasibility study.^69^  Blood will be collected at 4 weeks, and 8 weeks on study.

## 6.5 Concomitant Therapy

Subjects should continue all other medications, including pain medications, as used prior to enrolling in the study. All baseline medications, over the counter medications, herbals or supplements used will be collected at study enrollment.  As dronabinol may improve pain control resulting in subjects requiring less opioid use to treat pain, change in opioids used is a secondary endpoint.  Opioids will be reconciled with dispensing records from the New York Prescription Monitoring Program at baseline, week 4, and week 8. Opioids will be converted into OMEs. Patient’s own opioids will not be counted, only the study drug will be counted.

# 7 Study Intervention Discontinuation and Participant Discontinuation/Withdrawal

## 7.1 Discontinuation of Study Intervention

For serious adverse events (SAEs), defined as those which result in death, are life-threatening, require inpatient hospitalization or prolongation of existing hospitalization, result in significant disability or incapacity, or result in congenital anomaly or birth defect, Dr. Curtis will be notified and if the event is determined to be possibly related to dronabinol it will be discontinued.  Dronabinol will not be discontinued for SAEs determined to be unrelated to dronabinol, such as pain crisis. In the event that a participant withdraws from the study or discontinues dronabinol due to a SAE, the participant’s ongoing status will be monitored by Dr. Curtis until: 1) a resolution is reached, i.e., the problem requiring hospitalization has resolved or stabilized with no further changes anticipated; 2) the SAE is determined to be clearly unrelated to study intervention; or 3) the SAE results in death. In cases where a participant discontinues dronabinol for any reason, the Study

 Coordinator will ask the participant to continue to attend all study visits and complete assessments. Criteria for discontinuation of the randomization condition include 1) pregnancy; 2) current suicidal ideation with plan or intent; 3) suicide attempt; 4) severe anxiety episode, severe depression episode, or psychotic symptoms; 5) severe adverse events probably or definitely related to the randomization condition, as defined above; 6) adverse events that have the potential to cause serious impairment of health, including ischemic or arrhythmic adverse cardiovascular disease events (e.g., myocardial infarction, unstable angina, coronary revascularization, coronary artery disease, unstable arrhythmias, transient ischemic attack, stroke, or congestive heart failure); 7) serious adverse events, defined as those which result in death, are life threatening, require inpatient hospitalization or prolongation of existing hospitalization, result in significant disability or incapacity, or result in congenital anomaly or birth defect; or 8) the request of participants or their treating providers. Dr. Curtis or the Study Coordinator will be responsible for informing the participant to discontinue the randomization condition and will ask the participant to continue to attend all study visits. Once the randomization condition has been discontinued it will be for the duration of the study.

## 7.2 Participant Discontinuation/Withdrawal from the Study

Subjects may discontinue participation at any time by informing an investigator, but there is no plan to discard previously acquired data. Subjects experiencing study agent related adverse events will be encouraged to continue study follow-up until resolution, if feasible.

A subject’s participation may be discontinued at any time by any of the investigators if discontinuation is thought to be in the subject’s best interest, if continued participation is unlikely to yield further useful data due to noncompliance with study procedures, or if the study is discontinued.

## 7.3 Lost to Follow-Up

**Retention strategy.** We will employ several strategies to maximize retention:

1.  Subject tracking. At all visits, we will record: 1) subjects’ address, phone numbers; 2) contact information of family or friends; and 3) locations where subjects work.

2.  Engaging subjects. Frequent contact with study staff helps to keep subjects engaged. As we have done previously, we will contact subjects by phone if they miss a questionnaire. In addition, we will hire study staff who are racial and ethnic minorities consistent with our source population to enhance mutual understanding and engagement.

3.  Subject compensation. To increase feasibility of attending each visit subjects will be compensated $25 per office visit and a two way metro card for cost of travel. Subject compensation will occur through study debit cards.

**Estimates and monitoring for attrition.** In this study, we conservatively estimate attrition of 20%. Retention reports will be included in reports to the DSMC every 6 months.

**Benchmarks and contingency plans.** We will conduct assessments every 6 months to verify benchmarks of: 90% retention rate. If benchmarks are not met, will institute contingency plans such as: adding or reserving more physician time for evaluations, better coordination between research and clinical staff, revising the incentive structure, or making other modification based on reasons for attrition.

# 8 Study Assessments and Procedures

## 8.1 Efficacy Assessments

Informed consent will be obtained prior to screening activities. Screening may take multiple visits but must be completed within 30 days of obtaining informed consent. The following assessments will be completed:

- Written Informed consent
- Demographics
- Medical history - Including SCD type, SCD related problems, other medical problems, psychiatric and substance use history, description of pain and pain treatment
- Weight
- Vital Signs
- Review of current and past medications
- Pregnancy test - For women of child bearing potential Urine or serum.
- Urine test for cannabinoids
- Physical exam
- Baseline Patient Reported Outcomes (ASCQ-Me survey domains for pain impact, emotional impact, social impact, stiffness, and sleep, PROMIS domains for pain impact, neuropathic pain, nociceptive pain, cognitive function, gastrointestinal distress and anxiety. LANSS, PQ-B, C-SSRS)

Data will be collected from five sources: 1) Validated patient reported outcome surveys at baseline, and weeks 4 and weeks 8; 2) Timeline Follow-Back for hospital utilization use at research visits at weeks 4 and 8; 3) medical interview and physical examination at screening, weeks 4 and weeks 8; 4) laboratory markers of inflammation including IL1b, IL4, IL6, IL10, VCAM-1 and TNFα, tryptase, and substance P at baseline, weeks 4 and weeks 8 5) urine toxicology tests at baseline and weeks 4 and weeks 8, 6) safety laboratory tests of renal function, liver function, complete blood count and reticulocytes and pregnancy at screening and at weeks 4 and weeks 8;

In-person screening research interview, screening medical interview and physical examination data will be recorded on paper data collection instruments that identify participants only by unique study ID number. Patient reported outcome surveys will be performed on an I-Pad and entered automatically into REDCap. Data will be entered into a password-protected, encrypted web-based REDCap database, and paper documents will be filed in a locked cabinet.

Urine toxicology tests will be performed at baseline and blood tests for cannabinoids at weeks 4 and weeks 8. Specimens will be labeled with the name of the research study, the date and time of collection, and the specimen number; no names or other identifying information will be used. The specimen number will be generated using the subject’s study ID number, the letters UT (to denote urine toxicology test), and the visit number. The laboratory will run toxicology tests for THC, methadone, opiates, cocaine, barbiturates, PCP, amphetamines, alcohol, and benzodiazepines on each specimen. The laboratory results will be available to Dr. Curtis and the study assistant via the HIPAA compliant, encrypted, password protected clinical information system. They will not be reviewed until after the subject has completed the study to protect the masking.

We will conduct a blood test at screening and at weeks 4 and 8.  Laboratory markers of inflammation including IL1b, IL4, IL6, IL10, VCAM-1 and TNFα, tryptase, and substance P and safety laboratory tests of renal function, liver function, complete blood count and reticulocytes and pregnancy at screening and at weeks 4 and 8.

Assessments at weeks 2 and 6 are safety assessments only which consist of self reported adverse events, the PQ-B and the CSSR. These visits may be completed as in person or telemedicine visits.

## 8.2 Safety and Other Assessments

We have well-established protocols to screen for medical and psychiatric eligibility. Dr. Curtis conduct a thorough medical and psychiatric screening of potential participants who meet initial screening criteria and who provide informed consent. Eligibility criteria are detailed in Human Subjects and the determination of eligibility will include:

- Second screening visit. At the screening visit with Dr. Curtis potential participants will be further assessed for medical and psychiatric eligibility. A physical examination will be performed, women with reproductive potential will take a pregnancy test, and psychiatric disease and substance use disorders will be assessed using the following standardized instruments: 1) the Prodromal Questionaire -Brief (PD-B) a short structured interview to assess for psychotic symptoms; and 2) the Columbia Suicide Severity Rating Scale (CSSR).

Throughout the proposed study, Dr. Curtis will be responsible for continual medical oversight of patients. All participants will be given oral and written instructions about management of adverse medication effects and emergency contact information prior to initiating medication therapy.

Adverse events, including cardiovascular events, will be assessed at weeks 2, 4, 6, and 8 by asking participants about symptoms that emerged or worsened since the start of medication treatment, whether or not they were thought to be related to study medication. Participants will be asked about treatment-emergent symptoms using both a structured interview and open-ended questions. In the structured interview, we will assess for the presence of specific symptoms reported among ≥ 5 % of participants receiving dronabinol in published research, including (but not limited to) nausea, headache, anxiety, dizziness, paranoid thoughts, sleepiness, euphoria. We chose the threshold of 5% as the majority of published dronabinol studies did not report symptoms that occurred in <5% of participants. Treatment emergent psychiatric symptoms will be further assessed using structured psychometric instruments at each research visit (as described below). All documented adverse events will be documented in case report forms and followed up to resolution or end of study.

In order to protect patients from the potential psychiatric risks of dronabinol we developed and implemented detailed protocols for the assessment of incident psychiatric symptoms. Psychiatric symptoms and suicidal ideation will be assessed at weeks 2, 4, and 8, using the PD-B I and the CSSR. If participants meet diagnostic criteria for any new DSM-V Axis I diagnoses, or report suicidal ideation, it will trigger immediate clinical intervention.

We will conduct a blood test at screening then safety laboratory tests to evaluate for possible changes in renal function, liver function, complete blood count and reticulocytes and pregnancy at weeks 4 and 8.

## 8.3 Adverse Events and Serious Adverse Events

### 8.3.1 Definition of Adverse Events (AE)

An Adverse Event (AE) is any unfavorable and unintended sign (including an abnormal laboratory finding), symptom, or disease temporally associated with the use of a medical treatment or procedure that may or may not be considered related to the medical treatment or procedure. An AE is a term that is a unique representation of a specific event used for medical documentation and scientific analyses.

All subjects will be monitored for AEs during the study. Assessments will be made by monitoring the following parameters: the subject’s clinical symptoms, laboratory, pathological, radiological or surgical findings, physical examination findings, or findings from other tests and/or procedures.

Any worsening (i.e., any clinically significant adverse change in the frequency or intensity of a preexisting condition) should be considered an AE. A diagnosis or syndrome should be recorded in the electronic case report forms (eCRFs) rather than the individual signs or symptoms of the diagnosis or syndrome.

Abuse, withdrawal, sensitivity or toxicity to dronabinol should be reported as an AE. Overdose, accidental or intentional, whether or not it is associated with an AE should be reported. Any sequela of an accidental or intentional overdose of study agent should be reported as an AE on the AE eCRF. If the sequela of an overdose is an SAE, then the sequela must be reported on an SAE report form and on the AE eCRF. The overdose resulting in the SAE should be identified as the cause of the event on the SAE report form and eCRF but should not be reported as an SAE itself.

All AEs will be recorded by study personnel from the time the subject signs informed consent until 28 days after the last dose of dronabinol/placebo or until the final visit, whichever is longer, as well as those SAEs made known to the investigator at any time thereafter that are suspected of being related to study agent. Adverse events and SAEs will be recorded on the AE page of the eCRF.

Though dronabinol is an FDA approved medication with a known safety profile surveillance will occur for both known and unknown adverse events.

All laboratory abnormalities will be evaluated by the investigator or a qualified designee and recorded in the eCRF.

Regardless of severity grade, only laboratory abnormalities that fulfill a seriousness criterion need to be documented as a SAE.

If a laboratory abnormality is one component of a diagnosis or syndrome, then only the diagnosis or syndrome should be recorded on the AE page/screen of the eCRF. If the abnormality was not a part of a diagnosis or syndrome, then the laboratory abnormality should be recorded as the AE. If possible, the laboratory abnormality should be recorded as a medical term and not simply as an abnormal laboratory result (e.g., record thrombocytopenia rather than decreased platelets).

### 8.3.2 Definition of Serious Adverse Events (SAE)

An adverse event (AE) or suspected adverse reaction is considered "serious" if, in the view of the investigator, it results in any of the following outcomes:

- Results in death
- Is life-threatening (i.e., in the opinion of the investigator, the subject is at immediate risk of death from the AE)
- Requires inpatient hospitalization or prolongation of existing hospitalization (hospitalization is defined as an inpatient admission, regardless of length of stay)
- Results in persistent or significant disability/incapacity (a substantial disruption of the subject’s ability to conduct normal life functions)
- Is a congenital anomaly/birth defect
- Constitutes an important medical Important medical events are defined as those occurrences that may not be immediately life threatening or result in death, hospitalization, or disability, but may jeopardize the subject or require medical or surgical intervention to prevent one of the other outcomes listed above. Medical and scientific judgment should be exercised in deciding whether such an AE should be considered serious.

For this study, events not considered to be SAEs are hospitalizations for:

- Routine treatment or monitoring not associated with any deterioration in condition.
- The administration of blood or platelet transfusion as routine treatment of studied
- Emergency outpatient treatment or observation that does not result in admission, unless fulfilling other seriousness criteria
- Hospitalization for treatment of uncomplicated sickle cell pain crisis as defined by the investigator

If an AE is considered serious, both the AE eCRF and the SAE Report Form must be completed. For each SAE, the investigator will provide information on severity, start and stop dates, relationship to study agent, action taken regarding study agent, and outcome.

The principal investigator is responsible for reporting SAEs to the IRB and other applicable regulatory authority. The principal investigator is responsible for submitting follow-up reports for all SAEs regarding the patient’s subsequent course until the SAE has resolved or until the patient’s condition stabilizes (in the case of persistent impairment), or the death of the patient.

### 8.3.3 Classification of an Adverse Event

#### 8.3.3.1 Severity of Event

The severity of AEs will be graded and reported using the NCI Common Terminology Criteria for Adverse Events (CTCAE) v5.0. The full text of the NCI CTCAE is available online at: https://evs.nci.nih.gov/ftp1/CTCAE/CTCAE_5.0/

For adverse events (AEs) not included in the protocol defined grading system, the following guidelines will be used to describe severity.

- Grade 1: Mild – Events require minimal or no treatment and do not interfere with the participant’s daily
- Grade 2: Moderate – Events result in a low level of inconvenience or concern with the therapeutic measures. Moderate events may cause some interference with
- Grade 3: Severe – Events interrupt a participant’s usual daily activity and may require systemic drug therapy or other Severe events are usually potentially life- threatening or incapacitating. Of note, the term “severe” does not necessarily equate to “serious”.
- Grade 4: Life-threatening - Events resulting in a potential threat to life; urgent intervention extreme limitation in activity, significant assistance required; significant medical intervention/therapy required, hospitalization or hospice care probable
- Grade 5: Fatal - Event results in death related to AE

The distinction between the seriousness and the severity of an AE should be noted. Severity is a measure of intensity; thus, a severe reaction is not necessarily a serious reaction. For example, a headache may be severe in intensity, but would not be classified as serious unless it met one of the criteria for serious events

#### 8.3.3.2 Relationship to Study Intervention

All adverse events (AEs) must have their relationship to study intervention assessed by the clinician who examines and evaluates the participant based on temporal relationship and his/her clinical judgment. The degree of certainty about causality will be graded using the categories below. In a clinical trial, the study product must always be suspect.

- Definitely Related – There is clear evidence to suggest a causal relationship, and other possible contributing factors can be ruled The clinical event, including an abnormal laboratory test result, occurs in a plausible time relationship to study intervention administration and cannot be explained by concurrent disease or other drugs or chemicals. The response to withdrawal of the study intervention (de-challenge) should be clinically plausible. The event must be pharmacologically or phenomenologically definitive, with use of a satisfactory re-challenge procedure if necessary.
- Probably Related – There is evidence to suggest a causal relationship, and the influence of other factors is The clinical event, including an abnormal laboratory test result, occurs within a reasonable time after administration of the study intervention, is unlikely to be attributed to concurrent disease or other drugs or chemicals, and follows a clinically reasonable response on withdrawal (de-challenge). Re-challenge information is not required to fulfill this definition.
- Potentially Related – There is some evidence to suggest a causal relationship (e.g., the event occurred within a reasonable time after administration of the trial medication). However, other factors may have contributed to the event (e.g., the participant’s clinical condition, other concomitant events). Although an AE may rate only as “possibly related” soon after discovery, it can be flagged as requiring more information and later be upgraded to “probably related” or “definitely related”, as
- Unlikely to be related – A clinical event, including an abnormal laboratory test result, whose temporal relationship to study intervention administration makes a causal relationship improbable (e.g., the event did not occur within a reasonable time after administration of the study intervention) and in which other drugs or chemicals or underlying disease provides plausible explanations (e.g., the participant’s clinical condition, other concomitant treatments).
- Not Related – The AE is completely independent of study intervention administration, and/or evidence exists that the event is definitely related to another There must be an alternative, definitive etiology documented by the clinician.

Causality should be assessed and provided for every AE/SAE based on currently approved drug label. Causality is to be reassessed and provided as additional information becomes available.

#### 8.3.3.3 Expectedness

The PI or Sub-Investigator will be responsible for determining whether an adverse event (AE) is expected or unexpected. An AE will be considered unexpected if the nature, severity, or frequency of the event is not consistent with the risk information previously described for the study intervention and in the currently approved drug label.

### 8.3.4 Time Period and Frequency for Event Assessment and Follow-Up

All AEs will be recorded by study personnel from the time the subject signs informed consent until 28 days after the last dose of dronabinol/placebo or until the final visit, whichever is longer, as well as those SAEs made known to the investigator at any time thereafter that are suspected of being related to study agent.

### 8.3.5 Adverse Event Reporting

The reporting of AEs and SAEs will start when the patient signs the informed consent for the study throughout the treatment period and up to and including the 28-day follow-up period. All new AEs occurring during that period must be recorded (if SAEs, they must be reported to the Data Safety Monitoring Committee (DSMC) and IRB as per institutional guidelines). AEs will be recorded per SOPs.

The investigator will report the action taken with study agent as a result of an AE or SAE, as applicable (e.g., discontinuation, interruption, or dose reduction of study agent, as appropriate) and report if concomitant and/or additional treatments were given for the event.

- Patients receiving treatment with dronabinol capsules should be alerted to the potential for additive central nervous system depression if dronabinol capsules is used concomitantly with alcohol or other CNS depressants such as benzodiazepines and barbiturates.

Study agent will be discontinued for all AEs grade 3 or above. Study agent will not restarted until AE has resolved to grade 1 for all objective events or until patient grade 1 or grade 2 and patient requests to restart drug despite symptoms for all subjective events. This is because some adverse events of dronabinol may be seen as a benefit by patients, for example dronabinol may cause tiredness but some patients with SCD complain of insomnia.

Study agent will not be discontinued for expected AEs or SAEs determined to not be due to dronabinol such as a pain crisis.

For patient reported AEs grade 1 or 2 the patient and investigator will decide together whether to discontinue study agent, lower the dose of study agent, or to continue on full dose if the patient feels that the benefit of study agent outweighs the discomfort of the AE of the AE is in fact perceived as a benefit (i.e. sleepiness may be a benefit if the subject previously suffered from poor sleep).

The investigator will report the outcome of the event for both AEs and SAEs. All SAEs that have not resolved upon discontinuation of the subject’s participation in the study must be followed until recovered (returned to baseline), recovered with sequelae, or death (due to the SAE)

### 8.3.7 Reporting Events to Participants

### 8.3.8 Events of Special Interest

### 8.3.9 Reporting of Pregnancy

## 8.4 Unanticipated Problems

### 8.4.1 Definition of Unanticipated Problems (UP)

The Office for Human Research Protections (OHRP) considers unanticipated problems involving risks to participants or others to include, in general, any incident, experience, or outcome that meets all of the following criteria:

- Unexpected in terms of nature, severity, or frequency given (a) the research procedures that are described in the protocol-related documents, such as the Institutional Review Board (IRB)-approved research protocol and informed consent document; and (b) the characteristics of the participant population being studied;
- Related or possibly related to participation in the research (“possibly related” means there is a reasonable possibility that the incident, experience, or outcome may have been caused by the procedures involved in the research); and
- Suggests that the research places participants or others at a greater risk of harm (including physical, psychological, economic, or social harm) than was previously known

### 8.4.2 Unanticipated Problem Reporting

The investigator will report unexpected problems involving risks to subjects or others (UPIRSO) to the IRB per institutional SOPs.

### 8.4.3 Reporting Unanticipated Problems to Participants

# 9 Statistical Considerations

## 9.1 Statistical Hypotheses

- Primary Efficacy Endpoint(s):

Hypothesis: Participants who receive dronabinol will have an improvement in pain impact (primary outcome) at 8 weeks compared to patients who receive placebo.

- Secondary Efficacy Endpoint(s):

Hypothesis: Participants who receive dronabinol will have improvements in other measures of pain (average daily opioid use, neuropathic and nociceptive pain severity, health care utilization, and self-reported episodes of pain crisis) and quality of life (stiffness, sleep, emotional impact, and social impact) at 8 weeks compared to participants who receive placebo.

Hypothesis: Patients who receive dronabinol will have an increase in anti-inflammatory markers (IL4, IL10) reduction in pro-inflammatory markers (all others) at 8 weeks compared to patients who receive placebo.

Hypothesis: Participants in the dronabinol group will report greater incidence of low-grade adverse events such as dizziness or nausea but no greater serious AEs (cognitive function) compared to participants in the placebo group.

## 9.2 Sample Size Determination

In a study examining the responsiveness of PROMIS pain impact to changes in chronic pain patients who stated pain was a “little better” had an average increase of 1.8 point with a SD of 6.6 points and “much better” was associated with an average increase of 6.8 points with a SD of 6.5 points.^65^  Based on this we have chosen a change of 5 points as representing a clinically meaningful improvement in chronic pain, with an estimated a SD of 6.5 points. With a sample size of 28 patients (56 total) in each group we will have 80% power to detect a difference of 5 points in change between two groups in pain impact score from baseline at a two-sided 0.05 significance level.

We have a strong track record of following subjects with chronic pain, opioid use, and SCD in ourprior SCD studies have achieved 85-100% retention at 6 months. In our pilot trial we approached 27 patients to enroll 6 on study, we therefore estimate if we approach 280 patients, we can enroll 70. We conservatively estimate a 20% attrition rate, so we will aim to approach 280 patients to enroll 70 to ensure 56 participants completing the study.

## 9.3 Populations for Analyses

Primary analysis of all endpoints will be an intention to treat analysis.  Secondary analysis will be done removing patients who do not complete at least 4 weeks on study as well as any patients whose urine samples show presence of cannabinoid metabolites other than THC or whose pill counts reveal they took less than 50% of the reported optimized dose.   Safety analysis will be done on all patients who took at least one dose of the study medication.

## 9.4 Statistical Analyses

### 9.4.1 General Approach

We will first conduct descriptive and graphical analyses to check for outlier and abnormal values, verify that distribution of measures meets assumptions of planned statistical tests, and explore the success of randomization. Primary analyses for all aims will be intent-to-treat analyses of data from baseline to week 8, based on group allocation. Secondary analysis will be done removing patients who do not complete at least 4 weeks on study as well as any patients whose urine samples show presence of cannabinoid metabolites other than THC or whose pill counts reveal they took less than 50% of the reported optimized dose.

Statistical analysis will be done assuming all data is non-normal. Continuous data will be described as means and quartiles, categorical data will be described as percentages.  All inferential tests will be two-tailed.

### 9.4.2 Analysis of the Primary Efficacy Endpoint(s)

We will examine change in pain and quality of life measures from each participant’s own baseline after 8 weeks of treatment (difference in differences analysis). Median change from baseline of patients on dronabinol will be compared to median change from baseline of patients on placebo. We have chosen to compare change from baseline between the two groups to account for heterogeneity in cause and severity of chronic pain at baseline seen in people with SCD. Comparisons of change from baseline to end of study between patients on placebo and dronabinol will be done using a Rank Sum test. A backward stepwise multivariate regression analysis will be performed to assess for covariates associated with the primary and secondary outcomes.  As the group size will be small a regression analysis will also be done to assess if any covariates associated with the outcomes are significantly difference between the study groups.

### 9.4.3 Analysis of the Secondary Endpoint(s)

To account for inter-patient heterogeneity, our primary comparison will be to use a difference in differences analysis to examine change from their own baseline after 8 weeks of treatment with dronabinol. Comparisons of changes in WBC with differential, CRP, tryptase, substance P, and cytokines from baseline to end of study between patients on placebo and dronabinol will be done using t-tests or Wilcoxon rank-sum tests. A backward stepwise multivariate regression analysis will be performed to assess for covariates associated with the primary and secondary outcomes. As the group size will be small a regression analysis will also be done to assess if any covariates associated with the outcomes are significantly difference between the study groups.

### 9.4.4 Safety Analyses

Patients with SCD will be expected to have laboratory abnormalities and diagnoses of clinical or psychiatric conditions at baseline.  Comparison will be of new adverse events, complaints, or diagnosis in those on dronabinol to those on placebo.  The number and the percentages of patients experiencing new adverse events will be tabulated by study arm. Comparisons will be made using Fisher’s exact tests for each individual adverse event reported when appropriate.

### 9.4.5 Baseline Descriptive Statistics

Intervention groups will be compared on baseline characteristics, including demographics and laboratory measurements, using descriptive statistics.

Though we expect to balance important potential confounders of prior history of cannabis use and neuropathic pain between the two groups via randomization, we will examine whether these differ by randomization group and baseline or over the intervention period. If there are differences between groups, we will include these variables in models.

### 9.4.6 Planned Interim Analyses

Interim analyses of the data will be conducted when 50% of the sample has accrued. If the results show overwhelming, statistically significant differences in pain impactor opioid use between groups, the study will be stopped. If we find that serious adverse events are significantly more likely in the intervention arm the study will be stopped.

Interim analyses will be conducted by the biostatistician. Dr. Curtis will review all pertinent data to determine whether to continue accrual. A decision to stop the study may be made at any time that the research team and DSMC agree that an unacceptable type and/or frequency of adverse events has been observed. Dr. Curtis will report the decision to terminate the study to the DSMC and IRB within 48 hours of this determination. He will submit a narrative description of the reasons for early termination of the study to the IRB within 10 days.

### 9.4.7 Sub-Group Analyses

Exploratory secondary analysis will be done examining patients with neuropathic pain only, and other pain types only, and patients who were cannabinoid naïve vs previous cannabinoid users, though these may be underpowered.

### 9.4.8 Tabulation of Individual Participant Data

Individual participant data will be listed by measure and timepoint.

# 10 Supporting Documentation and Operational Considerations

## 10.1 Regulatory, Ethical, and Study Oversight Considerations

### 10.1.1 Informed Consent Process

#### 10.1.1.1 Consent/assent and Other Informational Documents Provided to participants

#### 10.1.1.2 Consent Procedures and Documentation

### 10.1.2 Study Discontinuation and Closure

### 10.1.3 Confidentiality and Privacy

### 10.1.4 Future Use of Stored Specimens and Data

### 10.1.5 Key Roles and Study Governance

| **Principal Investigator** | **Medical Monitor** |
| --- | --- |
|  |  |
|  |  |
|  |  |
|  |  |
|  |  |

### 10.1.6 Safety Oversight

### 10.1.7 Clinical Monitoring

### 10.1.8 Quality Assurance and Quality Control

### 10.1.9 Data Handling and Record Keeping

#### 10.1.9.1 Data Collection and Management Responsibilities

#### 10.1.9.2 Study Records Retention

### 10.1.10 Protocol Deviations

### 10.1.11 Publication and Data Sharing Policy

### 10.1.12 Conflict of Interest Policy

## 10.2 Additional Considerations

## 10.3 Abbreviations

| AE | Adverse Event |
| --- | --- |
| ANCOVA | Analysis of Covariance |
| ASCQ-Me | Adult Sickle Cell Quality of Life Measurement Information System |
| CBD | Cannabidiol |
| CFR | Code of Federal Regulations |
| CLIA | Clinical Laboratory Improvement Amendments |
| CMP | Clinical Monitoring Plan |
| COC | Certificate of Confidentiality |
| CONSORT | Consolidated Standards of Reporting Trials |
| CRF | Case Report Form |
| C-SSRS | Columbia Suicide Severity Rating Scale |
| DCC | Data Coordinating Center |
| DHHS | Department of Health and Human Services |
| DSMB | Data Safety Monitoring Board |
| DRE | Disease-Related Event |
| EC | Ethics Committee |
| eCRF | Electronic Case Report Forms |
| FDA | Food and Drug Administration |
| FDAAA | Food and Drug Administration Amendments Act of 2007 |
| FFR | Federal Financial Report |
| GCP | Good Clinical Practice |
| GLP | Good Laboratory Practices |
| GMP | Good Manufacturing Practices |
| GWAS | Genome-Wide Association Studies |
| HIPAA | Health Insurance Portability and Accountability Act |
| IB | Investigator’s Brochure |
| ICH | International Conference on Harmonisation |
| ICMJE | International Committee of Medical Journal Editors |
| IDE | Investigational Device Exemption |
| IND | Investigational New Drug Application |
| IRB | Institutional Review Board |
| ISM | Independent Safety Monitor |
| ISO | International Organization for Standardization |
| ITT | Intention-To-Treat |
| LSMEANS | Least-squares Means |
| LANSS | Leed’s Assessment of Neuropathic Symptoms and Signs |
| MedDRA | Medical Dictionary for Regulatory Activities |
| MOP | Manual of Procedures |
| MSDS | Material Safety Data Sheet |
| NCT | National Clinical Trial |
| NIH | National Institutes of Health |
| NIH IC | NIH Institute or Center |
| OHRP | Office for Human Research Protections |
| OME | Oral Morphine Equivalents |
| PI | Principal Investigator |
| PQ-B | Prodromal Questionnaire Brief |
| PROMIS | Patient Reported Outcomes Measurement Information System |
| QA | Quality Assurance |
| QC | Quality Control |
| SAE | Serious Adverse Event |
| SAP | Statistical Analysis Plan |
| SCD | Sickle Cell Disease |
| SMC | Safety Monitoring Committee |
| SOA | Schedule of Activities |
| SOC | System Organ Class |
| SOP | Standard Operating Procedure |
| THC | Tetrahydrocannabinol |
| UP | Unanticipated Problem |
| US | United States |

## 10.4 Protocol Amendment History

| **Version** | **Date** | **Description of Change** | **Brief Rationale** |
| --- | --- | --- | --- |
|  |  |  |  |
|  |  |  |  |
|  |  |  |  |
|  |  |  |  |
|  |  |  |  |
|  |  |  |  |
|  |  |  |  |
|  |  |  |  |
|  |  |  |  |
|  |  |  |  |
|  |  |  |  |
|  |  |  |  |
|  |  |  |  |
|  |  |  |  |
|  |  |  |  |
|  |  |  |  |
|  |  |  |  |
|  |  |  |  |
|  |  |  |  |
|  |  |  |  |
|  |  |  |  |
|  |  |  |  |
|  |  |  |  |
|  |  |  |  |

# 11 References
